# Deaths in London

**Published:** 1846-10

**Authors:** 


*Deaths in London.*—The deaths in London during the week ending Aug. 8th, amounted to 1133, 237 above the weekly average for the last five summers. Of these 136 were from pulmonary consumption.

